# Generation of supercoils in nicked and gapped DNA drives DNA unknotting and postreplicative decatenation

**DOI:** 10.1093/nar/gkv683

**Published:** 2015-07-06

**Authors:** Dusan Racko, Fabrizio Benedetti, Julien Dorier, Yannis Burnier, Andrzej Stasiak

**Affiliations:** 1 Center for Integrative Genomics, University of Lausanne, 1015-Lausanne, Switzerland; 2 SIB Swiss Institute of Bioinformatics, 1015-Lausanne, Switzerland; 3 Polymer Institute of the Slovak Academy of Sciences, 842 36 Bratislava, Slovakia; 4 Vital-IT, SIB Swiss Institute of Bioinformatics, 1015-Lausanne, Switzerland; 5 Institute of Theoretical Physics, École Polytechnique Fédérale de Lausanne (EPFL), 1015-Lausanne, Switzerland

## Abstract

Due to the helical structure of DNA the process of DNA replication is topologically complex. Freshly replicated DNA molecules are catenated with each other and are frequently knotted. For proper functioning of DNA it is necessary to remove all of these entanglements. This is done by DNA topoisomerases that pass DNA segments through each other. However, it has been a riddle how DNA topoisomerases select the sites of their action. In highly crowded DNA in living cells random passages between contacting segments would only increase the extent of entanglement. Using molecular dynamics simulations we observed that in actively supercoiled DNA molecules the entanglements resulting from DNA knotting or catenation spontaneously approach sites of nicks and gaps in the DNA. Type I topoisomerases, that preferentially act at sites of nick and gaps, are thus naturally provided with DNA–DNA juxtapositions where a passage results in an error-free DNA unknotting or DNA decatenation.

## INTRODUCTION

DNA topoisomerases permit DNA segments to pass through each other. These passages are necessary to decatenate and unknot newly replicated circular DNA molecules (1,2). However, it is not yet known how in crowded DNA molecules with many juxtapositions of DNA segments DNA topoisomerases can specifically recognize these DNA–DNA juxtaposition where passages lead to decatenation or unknotting rather than to creation of more complex entanglements (3–5). Several studies have shown that in relatively short DNA molecules the DNA–DNA juxtapositions resulting from knotting and/or catenation have, on average, slightly different geometry than other DNA–DNA juxtapositions (6,7). These differences may be sufficient to target action of DNA topoisomerases to catenated or knotted portions of DNA molecules and thus cause their selective decatenation or unknotting (2,8,9). However, *in vitro* studies using non-supercoiled DNA molecules have revealed that preferential DNA unknotting and decatenation by type II DNA topoisomerases is only efficient in the case of relatively short DNA molecules (4). Here we propose a radically different mechanism of selective DNA unknotting and decatenation. Using molecular dynamics simulations, we show that supercoiling generated in freshly replicated DNA molecules has the ability to push and confine DNA knots in the vicinity of single-stranded gaps and nicks where torsional stress is released. Nicks and especially gaps, due to their increased flexibility and reduced electrostatic repulsion, form then specific juxtapositions with other portions of the tightened knot. Such juxtapositions constitute preferred sites of DNA topoisomerase III action (10). Passages occurring at such juxtaposition lead then to very specific DNA unknotting and decatenation of freshly replicated DNA molecules.

DNA gyrase is bacterial type II DNA topoisomerase that uses the energy of ATP hydrolysis to introduce negative supercoiling into DNA (11,12). Negative supercoiling facilitates separation of DNA strands and as such eases the process of DNA replication and transcription (2). Studies using gyrase mutants and inhibitors revealed however multiple pleiotropic effects of gyrase deficiency with some of them having no obvious connection to increased energetic costs of strand separation (13). Among these effects are incomplete decatenation of freshly replicated bacterial chromosomes (14) and accumulation of knots in the DNA (15). Earlier studies addressing the effect of supercoiling on DNA unknotting considered ‘static’ situations where knotted molecules were already supercoiled to a physiological level (16). These studies did not consider though that DNA knots are mainly introduced during DNA replication (17,18) and that freshly replicated DNA molecules are initially torsionally unconstrained, as they contain short single-stranded gaps and nicks (19). However, gyrase acts on torsionally unconstrained DNA molecules (12) and induces in them axial rotation. Here we investigate the dynamic effects of arising supercoiling in knotted DNA molecules. To this aim, we introduced into modelled DNA molecules a motor consisting of an active swivel site where the elements flanking the swivel site are forced to continuously rotate with respect to each other (Supplementary Figure S1 and the methods section). Although the actual mechanism of DNA gyrase is in its details different from the active swiveling applied here, the end result is the same and consists of inducing relative axial rotation between two portions of the DNA that flank the active gyrase (12).

## MATERIALS AND METHODS

### Description of the model

Molecular dynamics simulations were performed using Extensible Simulation Package for Research on Soft Matter (ESPResSo) (20). We modified and extended the beaded chain model that has been used previously for simulations of DNA segregation in nanochannels (21) where modelled chains were subject to potentials aimed to reproduce tensile and bending flexibility of the DNA and excluded volume interactions. In the present study we included in addition dihedral potential aimed to reproduce torsional flexibility of DNA, soft repulsion potential mimicking effect of electrostatic repulsion and also considered hydrodynamic drag resulting from translational and rotational motion of the DNA in solution. We also introduced active swivels mimicking the action of DNA gyrase and passive swivels mimicking effect of nicks and single-stranded gaps in dsDNA.

### Generic chain model

The chain construction is presented in Supplementary Figure S1. Consecutive main chain beads (M) were bonded by a finitely extensible non-linear elastic potential (FENE) with the equilibrium bond length set to 1$\sigma$ (for the formula see Supplementary Figure S1). For modelled DNA molecules $\sigma$ = 2.5 nm and corresponds to ca 8 bp. The excluded volume interactions were accounted for by using shifted and cut fully repulsive Weeks–Chandler–Andersen (WCA) potential (22), so that for $r < \sqrt[6]{{2\sigma _{ex} }}$ the interaction energy $U_{{\rm WCA}} (r) = 4\varepsilon \left[ {\left( {\sigma _{ex} /r} \right)^{12} - \left( {\sigma _{ex} /r} \right)^6 + 0.25} \right]$ and $U_{{\rm WCA}} (r) = 0$ otherwise (23), $\varepsilon = k_B T$. The *r* represents center-to-center distance between two beads, $\sigma _{ex}$ was set to 1.5$\sigma$ to prevent intersegmental passages that were occurring sporadically in modelled supercoiled DNA molecules due to the action of the active swivel. Additionally, electrostatic repulsion was modelled as non-bonded interactions using Debye-Hückel approximation (24), $U_{{\rm DH}} (r) = \varepsilon l_{\rm B} q_i q_j /r{\rm exp(} - {\rm \kappa }r{\rm )}$ for *r* ≤ 8$\sigma$. The charge of individual beads was set to *q* = −2 as that closely corresponds to the remaining fraction of non-neutralized charges per 8 bp fragment in physiological solutions containing mono- and divalent counterions (25). The Bjerrum length (*l_B_*) for aqueous solutions was set to 0.71 nm = 0.28$\sigma$. The ${\rm \kappa }\;{\rm set}\;{\rm to}$1/(1.2$\sigma$) is the reciprocal Debye length. Molecular stiffness, or penalty for bending respectively, was introduced in the form $U_{\rm b} (\theta ) = K_{\rm b} (1 - \cos \theta )$, where *θ* is the angle formed by two consecutive principal bonds of the chain. The bending constant *K*_b_ was adjusted so that the chain stiffening resulting from the cumulative action of excluded volume, electrostatic repulsion and bending rigidity produced the chain with the persistence length of ca 50 nm. In order to introduce torsional constraint and also to mimic the rotational drag acting on DNA molecules in solution, we modified the classical beaded chain model by introducing periaxial beads (P) with no excluded volume but experiencing hydrodynamic drag (see Supplementary Figure S1). The periaxial beads (one per bond connecting main chain beads) were placed at distance of $\sigma$ = 1 from the main chain axis. The rigid bonds connecting auxiliary periaxial beads (P) with the auxiliary axial beads (A) placed in the middle between consecutive main chain beads (M) were maintained perpendicular to the main chain axis, similarly to our earlier study (26). The mass of each bead was set to *m*. This positioning of periaxial beads ensures that the dihedral angle between consecutive bonds connecting periaxial beads with the skeletal bonds of the chain is defined even for 90° bending between consecutive main bonds of the chain. The torsional constraint was introduced by dihedral angle potential acting on consecutive bonds connecting auxiliary periaxial beads (P) with auxiliary axial beads (A). The dihedral angle potential had the form *V*(*ϕ*) = *K*_D_[1 − cos (*ϕ* - *p*)], with the constant *K*_D_ = 20$\varepsilon$, the dihedral angle *ϕ* and the phase *p*. For generic regions of modelled DNA the phase *p* was set to 0.

### Models of DNA gyrase action, of DNA nicks and gaps and of topoIII action

To model the action of DNA gyrase, we selected one dihedral where the phase *p* was initially set to 0 but then was gradually changed during the simulation. To introduce a passive swivel mimicking the effect of a nick, the dihedral lock was interrupted between two consecutive beads. To mimic the effect of a short single-stranded gap, in addition to removing dihedral lock over a distance of three beads we also decreased by half the electrostatic charge per bead (since one bead represents then 8 bases and not 8 base pairs) and decreased the persistence length, since ssDNA is much more flexible than dsDNA (27). To model topoIII-mediated passages at the sites of DNA gaps we removed excluded volume interactions between gaps and the rest of modelled DNA molecules. This was sufficient to observe supercoiling-driven passages between gaps and duplex regions.

### Size and hydrodynamic drag of modelled DNA molecules

**Figure 1.**
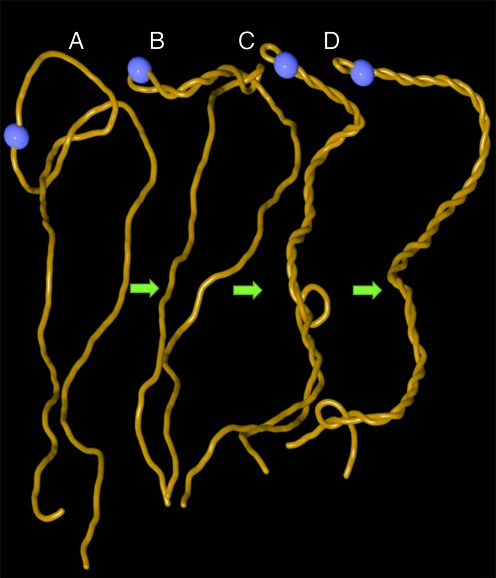
Arising supercoiling pushes trefoil knot out of the linear DNA. Snapshots from one continuous molecular dynamics simulation (see methods) where gyrase starts introducing negative supercoiling into knotted linear DNA that is ca 3 kb long. The site of active swivel (gyrase) is shown as the blue bead. (**A**) Starting configuration of knotted linear DNA molecule. (**B**) Formation of plectonemes starts in the vicinity of active gyrase. (**C, D)**. As plectonemically wound region increases it effectively pushes DNA segments that intervene with formation of relatively regular interwound regions. This process induces slithering of the knotted portion of the molecule towards the ends of knotted linear DNA and leads to its unknotting.

**Figure 2.**
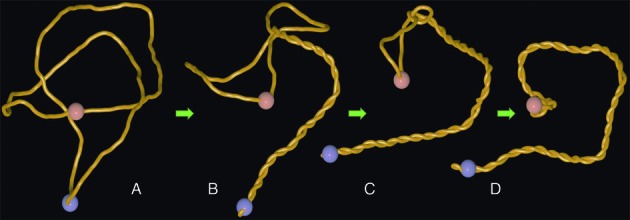
Arising supercoiling in nicked circular DNA pushes the trefoil knot towards the site of the nick. (**A**). A thermalized configuration of trefoil-forming nicked DNA molecule with ca 3000 bp with the sites of active swivel (gyrase) and passive swivel (nick) indicated as blue and reddish beads, respectively. (**B, C**) Working gyrase progressively confines the knotted portion and pushes it towards the site of the nick. (**D**) A snapshot from the steady state situation where the knot is localized in the vicinity of the nick and where the rate of supercoil generation by the gyrase is equal to the rate of supercoil dissipation at the site of the nick. Notice that knotted portion is highly localized and highly curved.

**Figure 3.**
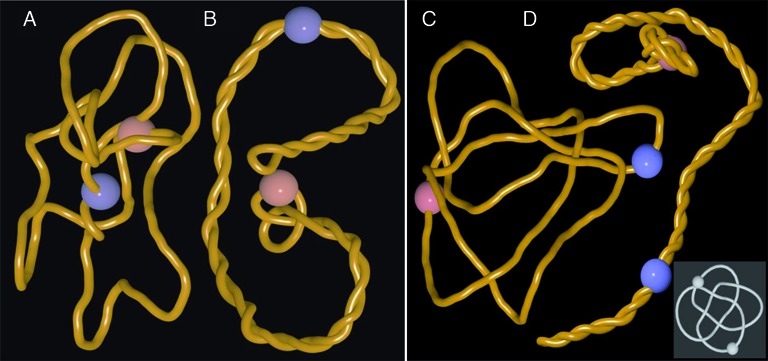
Pushing of DNA knots towards nicks and gaps does not require antipodal locations of active and passive swivel sites and it applies to all tested knot types. (**A, B)** snapshots of the simulation where active and passive swivel sites (indicated as blue and reddish beads, respectively) are placed 90° apart on the circular map of 3 kb large plasmid forming a trefoil knot. (**C, D)** snapshots from the simulation where a complex knot with 8 crossings (8_18_) is pushed towards the nick site. Notice that also in this case the knot is progressively tightened and localizes in the vicinity of a nick site (reddish bead). Inset shows 8_18_ knot in a form with its minimal number of perceived crossings.

**Figure 4.**
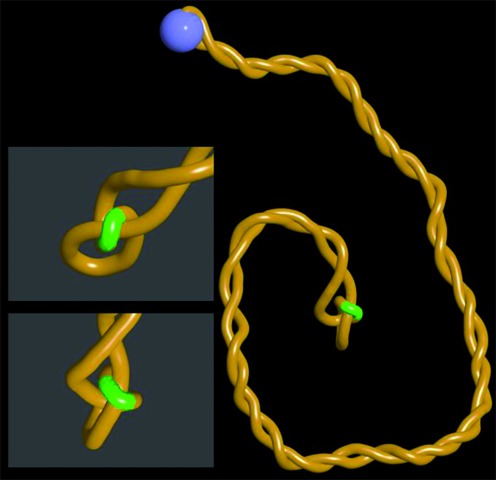
Juxtapositions between gaps and duplex regions in confined knots are perfectly suited for topoIII unknotting. Snapshots from the steady state situation where continuous rotations at the active swivel site (blue bead) generate supercoils confining and pushing the trefoil knot towards the site of a short gap (indicated as a green region). Notice on a close up views that gapped region bends over a duplex region located within the tightened knot. This specific structure results from the fact that single stranded DNA regions are very flexible and show decreased electrostatic repulsion since the number of charges in ssDNA is twice smaller than in dsDNA.

**Figure 5.**
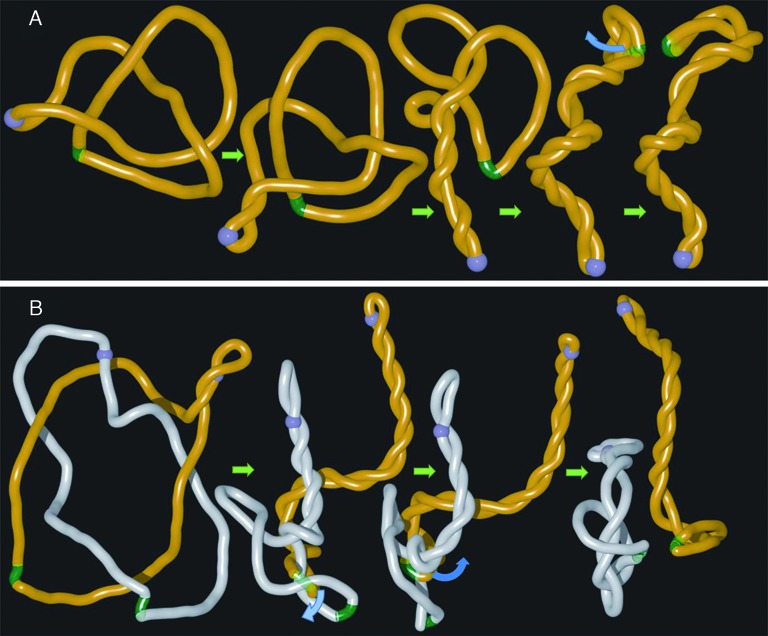
Model of DNA unknotting and decatenation involving long distance cooperation between DNA gyrase and topoIII. Simulation snapshots illustrating how gyrase (blue bead) that introduces DNA supercoiling and topoisomerase III acting at the site of gaps (green region) can act together in the process of DNA unknotting (**A**) and DNA decatenation (**B**). To simulate the process of topoIII mediated passages occurring at short gaps we have removed self-avoidance between gaps and the rest of modelled DNA molecules. Blue arcs in (A) and (B) indicate the directions of movements of double stranded regions that were allowed to pass through single stranded gaps as that would be the case of topoIII-mediated passages.

The size of modelled DNA molecules corresponded to 3000 bp in simulations presented in Figures 1–4 and to 1000 bp long DNA circles that were either knotted or catenated in simulations presented in Figure 5. To mimic hydrodynamic interactions experienced by DNA molecules subject to active supercoiling, the elementary units of the chain behaved as experiencing translational and rotational hydrodynamic drag opposing the rotational motion induced by modelled gyrase. The hydrodynamic drag *γ =* 0.5 *m*/$\tau$ for main skeletal beads (M) and to 0.2 *m*/$\tau$ for periaxial beads (P) and for auxiliary axial beads (A) between main skeletal beads (See Supplementary Figure S1). This setting of hydrodynamic drags results in a total hydrodynamic drag *γ* of 0.9 *m*/$\tau$ per repeating unit of our modelled chains. Usually used values of hydrodynamic drag for individual beads in beaded chains mimicking behaviour of DNA range between 0.5 and 1 *m*/$\tau$ (24,28).

### Calibration of simulation time units

The calibration of time unit $\tau$ was based on the comparison between simulated and experimentally determined rotational diffusion coefficient of DNA fragments with a size of 8 bp corresponding to repeating units of the model (29). In pure water, an 8 bp segment of DNA has a rotational diffusion coefficient of ca 6 × 10^7^ rad^2^/s. However, in living cell the viscosity experienced by DNA can be even 15 000 higher than this in pure water (30). Assuming that the diffusion coefficient is inversely proportional to viscosity (Stokes’ law), for a viscosity 5000 times higher than this of water the rotational diffusion coefficient will be 1.2 × 10^4^ rad^2^/s. In our simulations, the measured rotational diffusion coefficient is 1.6 rad^2^/$\tau$. To match the experimentally derived value of 1.2 × 10^4^ rad^2^/s, the time unit calibration results in $\tau$ = 1.3 × 10^−4^ s. The active swivel speed, which was set to 7 × 10^−4^ rotations per time unit, corresponds then to 5.2 turns per second, in agreement with experimentally observed swiveling speeds of DNA gyrase ranging between 1 and 5 rotations per second (12).

For simulations shown in Figure 4 due to small size of modelled DNA the diffusion and dissipation of supercoiling through the gaps was too fast to induce formation of plectonemic supercoils when the active swivel speed was set to 7 × 10^−4^ rotations per time unit of simulations. To induce formation of plectonemic supercoils in these unphysiologically short circular DNA molecules we increased the rotation speed to 7 × 10^−3^ rotations per time unit of simulations. It is possible that very short circular DNA molecules with nicks or gaps may not get supercoiled by ongoing action of one DNA gyrase per DNA molecule. Our simulations of these short circles were for the purpose of an illustration of what happens when supercoiling is created in knotted or catenated DNA molecules with short single-stranded gaps.

Within the simulations the Langevin equations of motion are integrated $m\ddot r_i = - \nabla U - \gamma \dot r_i + F_i^{\rm R}$, where $\gamma$ stands for the friction and it represents a drag of the environment (set to *γ* = 0.5 *m*/$\tau$ for individual skeletal beads and to 0.2 for periaxial beads and for middle points between the sequential skeletal beads) and where $F_i^{\rm R}$ is the random force. The setting of gamma in Langevin thermostat is 0.2, which determines the strength of the kicking by random force. The integration method for solving the equations of motion was the Verlet velocity integrator with a given time step Δ$\tau$ = 0.001. The average length of one simulation run was around 5 × 10^8^ integration steps.

## RESULTS

### Effect of supercoiling on linear DNA molecules with a knot

To investigate the effect of supercoiling generated in torsionally relaxed but knotted DNA molecules we studied first what happens to knotted linear DNA molecules when gyrase starts working as an active swivel. Figure 1 shows snapshots from molecular dynamics simulation where modelled DNA gyrase (indicated as a blue bead) progressively introduces negative supercoiling into linear DNA that contains a trefoil knot. Trefoil knots are the simplest and also most frequently observed knots in the DNA (31). Since in the viscous and crowded interior of bacterial cells the diffusion and dissipation of supercoils introduced by gyrase is relatively slow the resulting torsional stress causes formation of plectonemes (Figure 1B). As plectonemically interwound region grows, it effectively excludes the interwound portion from penetration by any intervening DNA fragments. This exclusion effect progressively pushes entanglements resulting from knotting towards the ends of linear DNA (Figure 1B–D) causing DNA unknotting (see also Supplementary Video 1).

### Effect of supercoiling on nicked circular DNA molecules with a knot

Next, we simulated the consequences of gyrase action on knotted circular plasmids containing nicks (modelled as passive swivels). This corresponds to the situation of freshly replicated plasmids that contain nicks and gaps in the terminator region (19) and which are known to be relatively frequently knotted (17,18). In the shown example (Figure 2 and Supplementary Video 2) the site of gyrase action (blue bead), is placed antipodally to the site of passive swivel (reddish bead) that provide the modelled DNA with the possibility of free swiveling just like it is the case of nicks and single-stranded gaps in real DNA. It is visible that as the gyrase starts working a plectonemic region forms in its vicinity (Figure 2B). The growing plectonemic region progressively confines the knotted region and pushes it towards the site of a passive swivel where supercoiling is continuously released. At the end of the simulation one obtains a steady state situation where the confined and strongly curved knotted portion of the molecule encompasses the passive swivel site (Figure 2D). In this steady state configuration the rate of generating supercoils by gyrase (active swivel) equals the rate of their release at the site of nick or gap (passive swivel).

Pushing of DNA entanglements towards nicks or gaps is not limited to cases where the sites of gyrase action are maximally distant from the sites where the supercoiling is released i.e. nicks or gaps. Practically the same phenomenon was observed when the active and passive swivel sites were present in the same quarter of modelled 3 kb plasmid (see Figure 3A and B). Pushing of entanglements resulting from knotting towards nicks or gaps is not limited to cases of simple knots such as trefoil knots. We observed this behaviour for all tested knots. Figure 3C and D as well as the Supplementary Video 3 show snapshots illustrating what happens when a much more complex knot such as 8_18_ is pushed by arising supercoiling towards nicks or gaps (inset in the figure presents a standard tabular representation of the knot 8_18_).

### Supercoiling of gapped DNA molecules with a knot predisposes them for topoIII unknotting

It is known that bacterial DNA topoisomerase III promotes decatenation and unknotting in DNA molecules by mediating passages of duplex regions through short single stranded gaps (10,19). We therefore simulated what happens when the site of passive swivel has properties of a short gap. Since gaps are single stranded the local electrostatic charge is reduced there by 50%. Single stranded gaps are also much more flexible than double-stranded regions (27,32). Figure 4 and Supplementary Video 4 show snapshots of the steady state situation where arising supercoiling pushed the trefoil knot towards a gap. Magnified images of the knotted region show that the gap is located within the knotted portion and there is a close juxtaposition of the gap with other region of the confined knot. If one would allow a passage between the gap and the juxtaposed double-stranded region, just as topoisomerase III would do it, the knot would be unknotted. In addition, such a passage leading to unknotting would decrease the elastic energy of the DNA. Such an energy gradient is important to direct the action of topoIII since it belongs to type I DNA topoisomerases, which in contrast to type II topoisomerases do not have ATPase activity and therefore cannot act against the free energy gradient.

### Model of supercoiling-assisted topoIII-mediated DNA unknotting and decatenation

The presented results suggest a possible model of efficient mechanism of DNA unknotting and DNA decatenation. Figure 5A presents a model of DNA unknotting where DNA gyrase cooperates with topoisomerase III acting at distally located ssDNA–dsDNA juxtapositions at sites of short gaps. In the first stage of the process the knot is progressively confined and pushed by arising supercoiling towards the site of single-stranded gap. Due to reduced electrostatic repulsion and reduced rigidity of the single-stranded gap, the lowest energy state of highly confined knot is reached when the short gap forms a juxtaposition with duplex region within the knotted portion (see Figures 4 and 5A). Such ssDNA–dsDNA juxtapositions are the preferred sites of action of topoisomerase III (33). A passage occurring at such juxtapositions leads then to DNA unknotting. The images in Figure 5A presenting our model of DNA unknotting are in fact snapshots from one continuous simulation run, where to mimic the action of topoIII at ssDNA gap, we have removed mutual self-avoidance between beads representing the gap and these representing the rest of modelled dsDNA molecules (see Supplementary Video 5).

DNA unknotting is important for cells, however, of much more fundamental importance is very efficient decatenation of freshly replicated circular DNA molecules that end the replication forming torus type of catenanes with several interlinks (34). Since separation of freshly replicated DNA molecules is crucial for cell survival, there are several pathways assuring successful decatenation (35). One of these pathways depends on action of topoIII on catenated DNA molecules with short single stranded gaps (19,36). Figure 5B shows results of our simulation investigating the consequences of arising supercoiling in catenated DNA molecules with single stranded regions. We can see that arising supercoiling confines and pushes the interlinked region towards gaps. Similarly as it was the case of knotted DNA, gaps form then juxtapositions with duplex portions and topoIII-mediated passages occurring at these juxtapositions lead to progressive decatenation (see also Supplementary Video 6). It needs to be reminded here that freshly replicated or even still replicating DNA molecules contain nicks and single stranded gaps.

The confinement of interlinked region by supercoiling should also facilitate the main decatenation pathway involving the action of topoisomerase IV. Strong confinement is likely to induce formation of higher order supercoiling with favourable geometry of dsDNA–dsDNA juxtaposition for topoIV action (37).

## DISCUSSION

It should be mentioned here that earlier simulation studies revealed that in a ‘static’ situation, where knotted molecules were already supercoiled and where gyrase does not introduce additional supercoils, knotted portions of molecules get more confined than in non-supercoiled DNA (38). However, such confined knots in ‘static’ supercoiled DNA molecules can be anywhere within the molecules. In the dynamic situation presented here, the supercoiling is generated in DNA molecules that are initially torsionally unstressed. The generated supercoiling induces axial rotation of DNA and by formation of plectonemes progressively tightens the knotted or catenated portions of the DNA and pushes them towards sites where supercoiling is dissipated. The sites where supercoiling is dissipated are nicks and gaps but can also be the sites where topoisomerases are bound to regular duplex DNA and release there the torsional stress arising during transcription and replication (2). DNA topoisomerases located near nicks or gaps or releasing the torsional stress in regular duplex DNA are then spontaneously provided with DNA–DNA juxtapositions where passages lead to error-free unknotting or decatenation.

It is good to add here that the linking number change introduced by DNA gyrase is redistributed into the change of twist and the change of writhe. Only the writhing and more specifically formation of plectonemes is responsible for pushing the knots towards nicks and gaps.

As mentioned in the Material and Methods section, our simulations were performed under conditions corresponding to an upper range of physiologically observed viscosities (30). Under these conditions even protein-free DNA molecules, such as modelled by us, experience large rotational drag. Large rotational drag was needed to prevent rapid dissipation of supercoils at DNA ends or sites of nicks and gaps. With slowed down dissipation of supercoils we observed formation of plectonemes in simulated by us linear and nicked DNA molecules with an active swivel introducing 5 rotations per second. *In vivo* the rotational drag of DNA results not only from relatively large viscosity of cellular milieu but also from the fact that DNA is bound by various proteins that effectively increase the diameter of formed nucleoprotein complexes and hence increase the drag. *In vivo* experiments demonstrated that transcribing RNA polymerase, inducing ca 5 axial DNA rotations per second, is sufficient to induce formation of supercoils when another RNA polymerase is simply bound to DNA stabilizing there a DNA bend that provides a strong rotational drag (39). In our simulations we do not include bound proteins or stable DNA bends. To keep our modelling simple and robust, we maintained the generic, isotropic model of the DNA but increased the rotational drag of DNA by modelling the situation in the upper range of viscosities reported in living cells (30).

It is interesting to estimate how much time it may take *in vivo* to move knots and catenane crossings towards nicks and gaps for unknotting and decatenation, respectively. In simulated 3 kb large plasmids ca 100 rotations of DNA were usually needed to push the knot towards a nick. With DNA gyrase action resulting in ca 5 rotations per second (12) it will require ca 20 s to create a substrate for topoIII unknotting. In larger plasmids the distance over which the enatnglements are required to move will increase and also the required time. However, in larger plasmids as well as in entire chromosomes several gyrase molecules can work in parallel increasing the speed of plectoneme growth and thus the speed of knots movement. In addition, the nearest gap in catenated and knotted DNA molecules may be close to knotted regions even in large DNA molecules. Once the entanglement resulting from knotting or catenation is localized at the site of gap it can be unknotted by topoIII within a fraction of a second as determined in single molecule experiments (10).

Proposed by us models of supercoiling-assisted topoIII-mediated unknotting and decatenation seem to be attractive. Is there however an experimental evidence supporting these models? Shishido et al., showed that *Escherichia coli* cells devoid of gyrase activity accumulated knotted DNA plasmids suggesting that gyrase activity is needed for efficient DNA unknotting (40). Separate study addressing the question of postreplicative DNA decatenation have showed that *E. coli* cells without TopoI but having TopoIII as the only type I DNA topoisomerase require supercoiling activity of gyrase for chromosome segregation (41). The interplay between topoIII and gyrase in chromosome segregation was also confirmed by showing that overexpression of topoIII is needed to segregate chromosomes in cells without active DNA gyrase (42).

## Supplementary Material

SUPPLEMENTARY DATA
