# Supplementary material for: Generation of supercoils in nicked and gapped DNA drives DNA unknotting and postreplicative decatenation
Source: Nucleic Acids Res. 2015 Jul 6;43(15):7229–36. doi: 10.1093/nar/gkv683 (PMC4551925; doi:10.1093/nar/gkv683)
Supplement: SUPPLEMENTARY DATA [file supp_43_15_7229__index.html]

Generation of supercoils in nicked and gapped DNA drives DNA unknotting and postreplicative decatenation — Generation of supercoils in nicked and gapped DNA drives DNA unknotting and postreplicative decatenation — SUPPLEMENTARY DATA 

# Generation of supercoils in nicked and gapped DNA drives DNA unknotting and postreplicative decatenation

## SUPPLEMENTARY DATA

- SUPPLEMENTARY DATA
- SUPPLEMENTARY DATA
- SUPPLEMENTARY DATA
- SUPPLEMENTARY DATA
- SUPPLEMENTARY DATA
- SUPPLEMENTARY DATA
- SUPPLEMENTARY DATA
