# Supplementary material for: Generation of supercoils in nicked and gapped DNA drives DNA unknotting and postreplicative decatenation
Source: Nucleic Acids Res. 2015 Jul 6;43(15):7229–36. doi: 10.1093/nar/gkv683 (PMC4551925; doi:10.1093/nar/gkv683)
Supplement: SUPPLEMENTARY DATA [file supp_gkv683_nar-01351-f-2015-File007.pdf]

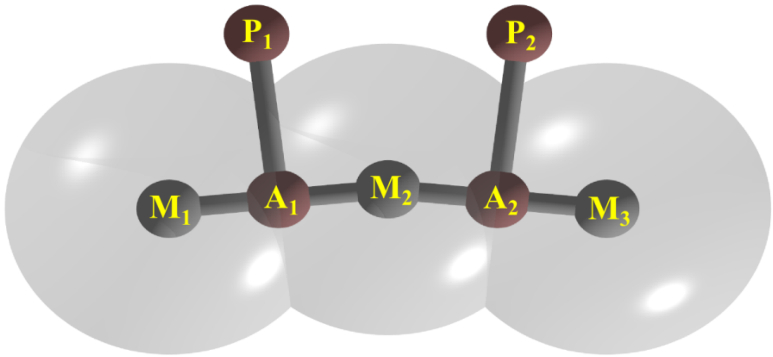

Bond potential	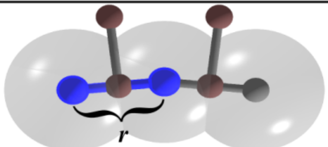	Bonds between: M_1M_2<br>$V(\boldsymbol{r}) = -0.5K\boldsymbol{r}_{\max}^2 \ln [1 - (\boldsymbol{r} - \boldsymbol{r}_0)^2 / \boldsymbol{r}_{\max}^2],$<br>$r_{\max} = 1.5\ \sigma,\ r_0 = \sigma,\ K = 30\ \varepsilon / \sigma^2$
	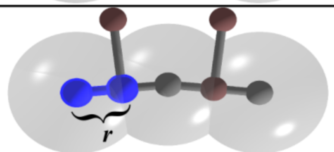	Bonds between: M_1A_1, M_2A_1, M_2A_2, M_3A_2<br>$V(\boldsymbol{r}) = k(\boldsymbol{r}-\boldsymbol{r_0})^2$, $k = 100\ \varepsilon$, $\boldsymbol{r_0} = 0.4\ \sigma$
	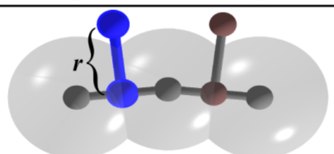	Bonds between: A_1P_1, A_2P_2<br>$V(\boldsymbol{r}) = k(\boldsymbol{r}-\boldsymbol{r_0})^2$, $k = 100\ \varepsilon$, $\boldsymbol{r_0} = \sigma$
Angular potential	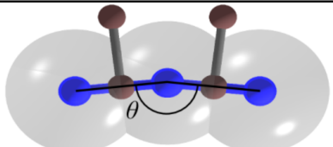	$\angle M_1M_2M_3$<br>$V(\theta) = K_b\ (1 - \cos\theta)$, $K_b = 8.5\ \varepsilon$
	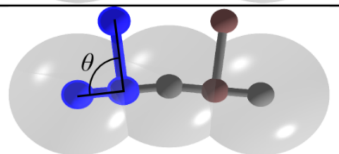	$\angle M_1A_1P_1$ $\angle M_2A_2P_2$<br>$V(\theta) = K_b\ [1 - (\cos(\theta-\theta_0)]$,<br>$K_b = 100\ \varepsilon$, $\theta_0 = \pi/2$
Dihedral potential	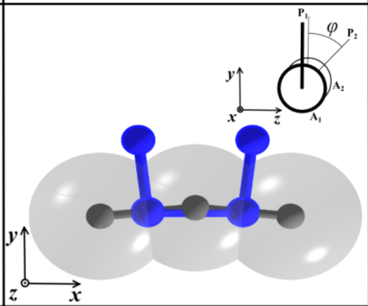	$\angle P_1A_1A_2P_2$ Generic chain:<br>$V(\varphi) = K_D(1- \cos\varphi)$, $K_D = 20\ \varepsilon$<br>Active swivel:<br>$V(\varphi) = K_D[1- \cos(\varphi - p)]$, $K_D = 20\ \varepsilon$<br>$p = (7 \times 10^{-4}\ .2\pi.t) / \tau$<br>Passive swivel:<br>$V(\varphi) = 0$
Pair potential	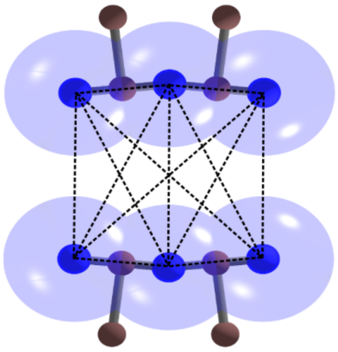	Excluded volume interaction, M_{ij}, $i \neq j$<br>$V(\boldsymbol{r}) = 4\varepsilon[(\sigma_{ex}/\boldsymbol{r})^{12}-(\sigma_{ex}/\boldsymbol{r})^6+1/4]$<br>$\boldsymbol{r} < 2^{1/6}\sigma_{ex}$, $\sigma_{ex} = 1.5\ \sigma$<br>Electrostatic interaction for all M_{ij}, $i \neq j$<br>$V(\boldsymbol{r}) = l_B k_B T Q_1 Q_2/\boldsymbol{r}\ \exp(-\kappa\ \boldsymbol{r})$<br>$\boldsymbol{r} < 8\ \sigma$, $l_B = 0.28$, $\kappa = 1/(1.2\ \sigma)$, $Q_i = -2$

Fig. S1. Construction of the worm-like chain model mimicking mechanical properties of DNA and its hydrodynamic interactions.

The chain is composed of main chain beads (M), with large zones of excluded volume shown as semitransparent spheres, and auxiliary beads that are needed to introduce torsional resistance via dihedral potentials as well as rotational hydrodynamic drag. There are two types of auxiliary beads: axial (A) and periaxial (P). The auxiliary beads have neither excluded volume nor electrostatic charge but are needed to introduce rotational drag mimicking rotational drag of DNA molecules. All bonding, angular, excluded volume and electrostatic potentials acting on modeled molecules are listed and specified together with drawings highlighting (in blue) the sets of beads and bonds involved in defining a given potential. Figure lists the relevant potentials for small portion of shown chain but the same applies to all consecutive generic portions of modeled chains. Dihedral potentials at active and passive swivels are indicated. In simulations shown in Fig. 4 the modelled single-stranded gaps acted as a set of consecutive passive swivels, had twice smaller electrostatic charge per bead than the modelled generic dsDNA and had no bending rigidity. In simulations shown in Fig. 5 gaps were in addition devoid of excluded volume interaction with respect to all beads representing double-stranded DNA regions. Gaps still exerted electrostatic repulsion on all other regions, which created a relatively small energy barrier opposing crossings between the gap and contacting duplex regions. This permitted us to mimic the action of topoIII that mediates passages between single stranded gaps and double-stranded DNA regions.
